# Hepatitis A Virus: Essential Knowledge and a Novel Identify-Isolate-Inform Tool for Frontline Healthcare Providers

**DOI:** 10.5811/westjem.2017.10.35983

**Published:** 2017-10-18

**Authors:** Kristi L. Koenig, Siri Shastry, Michael J. Burns

**Affiliations:** * Medical Director, EMS, County of San Diego, Health & Human Services Agency; † University of California Irvine, Department of Emergency Medicine, Orange, California; ‡ University of California Irvine School of Medicine, Department of Emergency Medicine, and Department of Medicine, Division of Infectious Diseases, Orange, California

## Abstract

Infection with hepatitis A virus (HAV) causes a highly contagious illness that can lead to serious morbidity and occasional mortality. Although the overall incidence of HAV has been declining since the introduction of the HAV vaccine, there have been an increasing number of outbreaks within the United States and elsewhere between 2016 and 2017. These outbreaks have had far-reaching consequences, with a large number of patients requiring hospitalization and several deaths. Accordingly, HAV is proving to present a renewed public health challenge. Through use of the “Identify-Isolate-Inform” tool as adapted for HAV, emergency physicians can become more familiar with the identification and management of patients presenting to the emergency department (ED) with exposure, infection, or risk of contracting disease. While it can be asymptomatic, HAV typically presents with a prodrome of fever, nausea/vomiting, and abdominal pain followed by jaundice. Healthcare providers should maintain strict standard precautions for all patients suspected of having HAV infection as well as contact precautions in special cases. Hand hygiene with soap and warm water should be emphasized, and affected patients should be counseled to avoid food preparation and close contact with vulnerable populations. Additionally, ED providers should offer post-exposure prophylaxis to exposed contacts and encourage vaccination as well as other preventive measures for at-risk individuals. ED personnel should inform local public health departments of any suspected case.

## INTRODUCTION

The incidence of hepatitis A virus (HAV) infection steadily decreased in the United States (U.S.) and other developed countries following the introduction of the HAV vaccine. Although vaccine became available in the U.S. in 1995, vaccination was not routinely recommended for children in California until 1999, and across the U.S. in 2006. The incidence of HAV decreased from six cases per 100,000 in 1999 to 0.4 cases per 100,000 in 2011.^1^,^2^ However, there has been a resurgence in the incidence of HAV in the U.S., with recent outbreaks occurring in San Diego, Los Angeles, New York City, Michigan, Hawaii, and several other counties and states. Between August 1, 2016, and October 12, 2017, there have been 397 confirmed cases of HAV in Michigan with 15 fatalities and 320 hospitalizations (85.6%). The Michigan Department of Health and Human Services has not yet identified a common source of the outbreak as of October 12, 2017.^3^ San Diego’s public health officer declared a local health emergency on September 1, 2017, due to the ongoing outbreak of HAV. As of October 17, 2017, the county has identified a total of 507 cases with 19 deaths and 351 (69%) hospitalizations related to the outbreak.^4^ Between June and October 2016, Hawaii reported 292 confirmed cases of HAV with 74 patients requiring hospitalization.^5^ This outbreak was linked to raw scallops served at a local sushi chain and a subsequent product recall was instated with no new cases reported as of July 11, 2017.^6^ A 2016 multistate outbreak of HAV linked to contaminated frozen strawberries resulted in 143 recognized cases and 56 hospitalizations.^7^

These HAV outbreaks pose a unique challenge for public health officials for several reasons: the prolonged incubation period (15–50 days); infected individuals can transmit the disease up to two weeks *prior* to symptom onset; many infected persons remain asymptomatic; and many patients affected in these outbreaks are homeless and/or illicit drug users (both injection and non-injection), causing difficulty in contacting and following up infected persons.^3^,^4^ Emergency Department (ED) providers in affected areas may encounter and treat a large number of these patients. Additionally, if the disease arises in other regions, it is likely that ED providers would be the first point of contact for many symptomatic patients. Given the contagious nature of HAV, as well as potential morbidity and mortality associated with the disease, it is of great importance that cases of the infection be accurately recognized, isolated and treated, with prompt notification of public health authorities. ED providers have a unique opportunity to advocate for vaccination of vulnerable populations, and EDs have enacted vaccination programs during acute outbreaks.^8^ After a thorough review of HAV infection, this paper describes a novel 3I tool, initially developed for Ebola virus and subsequently adapted for measles, MERS and mumps,^9^–^12^ for use by ED providers in the initial detection and management of HAV patients.

## CLINICAL PRESENTATION

HAV infection often presents with a prodromal period characterized by nausea, vomiting, anorexia, fever, malaise and abdominal pain. After a few days to weeks, patients may develop dark urine and pale, clay-colored stools as well as jaundice and pruritus. In some infected persons, there is no prodromal phase or it is so mild that the infected person does not present for medical care until jaundice develops. Approximately 70% of infected adults will exhibit initial symptoms; jaundice occurs in 40–70% of cases.^13^ During the prodromal phase, infected persons are highly contagious as there is viremia, and large quantities of infectious virus are shed in the stool. On physical examination, patients commonly present with fever, jaundice, scleral icterus and hepatomegaly.^14^,^15^ Less commonly, patients may demonstrate extrahepatic signs and symptoms of the disease, including splenomegaly, rash and arthralgias. In very rare cases, hematologic abnormalities (e.g. aplastic anemia, red cell aplasia and thrombocytopenia), neurologic abnormalities (e.g. optic neuritis and transverse myelitis), rheumatologic findings (e.g. leukocytoclastic vasculitis, glomerulonephritis, cryoglobulinemia) as well as toxic epidermal necrolysis and myocarditis can occur.^16^–^21^

**Population Health Research Capsule**

What do we already know about this issue?

Public health officials are reporting outbreaks of hepatitis A virus (HAV), the cause of a highly contagious illness that can lead to serious morbidity and occasional mortality.

What was the research question?

Investigators sought to modify the “Identify-Isolate-Inform” (3I) Tool for use in management of HAV outbreaks.

What was the major finding of the study?

A novel HAV 3I Tool is created for real-time application in managing patients presenting to the Emergency Department (ED).

How does this improve population health?

HAV presents a renewed public health challenge. ED providers have an essential role in assisting public health with management of this vaccine-preventable disease.

## RISK FACTORS

Populations at highest risk for HAV infection include travelers from high-income developed countries who visit endemic areas of Africa, Asia, and parts of Central and South America, men who have sex with men, close contacts (household or sexual) with infected persons, persons exposed to daycare centers, as well as the homeless, the incarcerated, and illicit drug users.^23^,^24^,^27^ In the 2016–17 Michigan and San Diego outbreaks in the U.S., half to three-quarters of infected individuals were homeless, recently incarcerated or illicit drug users.^3^,^22^ In the Hawaii outbreak from scallops and in the multistate outbreak from frozen strawberries, these populations were not at higher risk. Between December 2016 and June 2017, there has been an ongoing HAV outbreak in 20 European countries and Tel Aviv, Israel. As of September 27, 2017, 2,873 cases of HAV infection have been identified. 980 of these cases involved male patients. Of cases among male patients, 738 (76%) occurred among men having sex with men.^25^ Additionally, 17 cases of HAV in Tel Aviv have been linked to men having sex with men.^26^ Between January and August of 2017, there has also been an increase in HAV infection in men who have sex with men in New York City, with 46 identified patients as of September 22, 2017.^28^

## DIAGNOSIS

Clinically, HAV infection cannot be distinguished from other forms of viral hepatitis. Typically, the alanine aminotransferase level is very high, even in mild cases, including in the prodromal phase, usually approaching 1,000 units/L or greater, and is typically greater than the aspartate aminotransferase. Healthcare providers should suspect HAV infection in patients with the above-mentioned common symptoms (see “Clinical Presentation”), particularly in conjunction with elevated liver function tests. At initial presentation, persons with suspected viral hepatitis should have serologic testing for hepatitis A, B, and C, to include HAV immunoglobulin M (IgM), hepatitis B surface antigen, hepatitis B core IgM, and hepatitis C antibody. Serologic testing for HIV should be performed if HIV-status is not known. The prothrombin time/international normalized ratio should also be checked. Anti-HAV IgM is an indicator of acute infection and can be detected in blood for up to six months after infection. Anti-HAV IgG is indicative of either past infection or vaccination.^29^

## COMPLICATIONS AND SPECIAL POPULATIONS

Less than 1% of cases of HAV infection in adults will progress to fulminant liver failure. Within the U.S., only 3% of cases of liver failure in adults have been attributed to HAV infection.^30^,^31^ Among children in the U.S., HAV infection accounts for up to 1% of cases of acute liver failure.^32^ However, in countries with a higher disease incidence, some studies report that HAV infection accounts for up to 60% of pediatric liver failure cases.^33^,^34^ Patients with underlying liver disease, including those with chronic hepatitis B or C, are at greater risk for development of fulminant hepatic failure if they become infected with HAV infection.^35^ HAV-related acute liver failure has a spontaneous survival rate of approximately 69%. The remainder of individuals will either successfully undergo liver transplantation or progress to death.^31^ Management of acute liver failure due to HAV infection is similar to the management of liver failure due to other causes.

Unlike hepatitis B and C, HAV infection has no chronic carrier state and does not lead to chronic hepatitis or cirrhosis. Persons with chronic liver disease caused by hepatitis B or C who subsequently develop HAV infection may have increased morbidity and mortality.^36^ HAV infection can be complicated by the development of cholestatic hepatitis with a protracted period of jaundice. Clinical symptoms include jaundice, pruritus, fever, weight loss and diarrhea for a period of greater than three months. Laboratory tests will show elevated bilirubin, alkaline phosphatase and transaminitis. Cholestatic hepatitis will typically resolve without further intervention and treatment is limited to supportive management.^37^,^38^

Relapsing hepatitis can complicate some cases of HAV infection. A relapse of symptoms may occur a couple of weeks to several months after the original illness. Symptoms during relapse are typically milder in severity when compared to the initial acute illness. Treatment is focused on supportive care, and resolution typically occurs without further intervention.^39^ HAV can rarely lead to the development of autoimmune hepatitis that can have a prolonged and complicated clinical course.^40^

## TRANSMISSION AND PERSONAL PROTECTIVE EQUIPMENT

The fecal-oral route is the primary mechanism of transmission for HAV. Transmission typically occurs via close person-to-person contact (sexual or household) or via exposure to food or water contaminated by human feces, even in minute amounts. For all practical purposes, humans are the only host for the HAV virus. The incubation period for HAV is about 28 days on average, but can range from 15–50 days. Patients are considered contagious for both two weeks prior to, and up to 1–2 weeks after symptom onset.^41^ Rarely, the virus can be excreted in the stool for weeks to months, especially in immunocompromised children. The virus can be contracted from cooked food if the food is either not heated to an adequately high temperature (>185° F, >85° C) or if it is contaminated after being cooked.^43^ Healthcare providers treating potentially infected patients should observe standard precautions including using gloves and handwashing with soap and warm water. Importantly, HAV virus may not be inactivated by alcohol-based hand rubs.

Infectious virus may remain viable on surfaces for months and is resistant to many chemical agents, but is killed by household bleach (hypochlorite). Chlorine bleach solution should be used to disinfect frequently touched surfaces. Some outbreak cities have initiated power-washing of sidewalks and street areas with a bleach and chlorine solution in areas with a high density of homeless populations. Gown and gloves should be worn prior to disinfecting and cleaning affected areas. Further isolation measures are not routinely recommended.^45^,^46^

## DIFFERENTIAL DIAGNOSIS

The differential diagnosis for a patient presenting with symptoms of HAV virus infection is broad. It includes hepatitis B, C, D and E viruses. Other infections in which acute hepatitis sometimes manifests include Epstein-Barr virus, cytomegalovirus, yellow fever, disseminated herpes simplex, adenovirus, HIV infection, malaria, leptospirosis, syphilis, Rocky Mountain spotted fever, typhoid fever and Q fever.^47^ Non-infectious causes of similar symptoms should also be considered, including drug-induced liver injury (e.g., from acetaminophen), Budd-Chiari syndrome, Amanita phalloides mushroom poisoning, and autoimmune hepatitis.^48^

## TREATMENT

HAV is typically a self-limited infection and treatment is primarily directed towards supportive care, including analgesics, hydration and medication for pruritus. Patients should be instructed to avoid hepatotoxic medications and alcohol. In cases of acute liver failure due to HAV, transfer to a liver transplantation center should be considered. Other complications of HAV infection should be managed via a standard approach to those disease entities.^47^

## PREVENTION

The primary method of prevention of HAV infection is through vaccination. In the U.S., the vaccination is a two-dose series licensed for use in all individuals above the age of 12 months. The Centers for Disease Control and Prevention (CDC) recommends vaccination for the following: all children at one year of age; children and adolescents 2–18 years of age who live in areas with high disease incidence who have not been vaccinated at age one; persons traveling to or temporarily residing in developing countries with increased incidence of HAV; men who have sex with men; patients who use illegal drugs (both injection and non-injection); persons with occupational risk factors (persons who either work with HAV-infected primates or with HAV virus in a laboratory setting); persons with chronic liver disease or who have received/are awaiting liver transplants; persons with clotting-factor disorders; and close contacts of adopted children from countries with increased incidence of HAV infection. In the future homeless individuals may be added to the list of persons for whom vaccination is recommended.

The HAV vaccine is composed of an inactivated virus; accordingly, it is safe for administration to immunocompromised persons. The safety of the vaccine in pregnancy is indeterminate at this time (although thought to be low risk). A discussion of risks as well as benefits should be held with pregnant patients prior to administration of vaccination.

Persons with recent exposure to HAV can be administered the single agent HAV vaccine within two weeks of exposure to prevent infection. They should not be given the combined HAV/HBV vaccine as post-exposure prophylaxis (PEP) since a single dose of the combination may be less efficacious in inducing protective antibody. While the regular vaccination schedule requires an additional vaccine dose in six months, this may be impracticable in homeless and drug-using populations. For outbreak control, a single vaccination is effective and has an efficacy of 94–100% in adults and 97–100% in children.^49^ Intramuscular immune globulin (IG) can also be used for the same purpose. The IV formulation of IG should be not be used since it contains lower titers of protective antibody.

Immunocompromised patients, children aged less than 12 months, patients with chronic liver disease, and patients with an allergy to the vaccine or vaccine component should be treated with intramuscular IG at dose of 0.1 mL/kg. CDC recommends PEP with IG rather than vaccine for persons over age 40. Some public health departments (e.g., California Department of Public Health) recommend vaccine without IG through age 59, and vaccine plus IG for persons aged 60 and over. Persons administered IG should receive HAV vaccine concurrently if it is also recommended for other reasons. Immunocompromised persons may also be offered the vaccine in addition to IG, but vaccine response may be reduced. PEP is recommended for close personal contacts of infected individuals, unvaccinated staff members and attendees at affected child-care centers, and for persons exposed to food or water from a common infected source. Some ED and prehospital providers who are caring for high-risk populations during outbreaks have been offered vaccination. Even if unvaccinated, healthcare workers who manage a patient infected with HAV do not routinely require PEP as long as standard precautions and adequate hand hygiene are observed.^43^,^44^

## DISPOSITION

Due to the self-limiting nature of HAV infection, most immunocompetent patients without major comorbidities can be managed as outpatients with instructions to maintain good hand hygiene, avoidance of sharing of food or towels, and with supportive measures. Standard admission criteria should be used to assess symptomatic patients and determine whether hospitalization is required. As noted above, patients with acute hepatic failure due to HAV should be transferred to a liver transplantation center if feasible.

## IDENTIFY-ISOLATE-INFORM

The identify-isolate-inform tool, initially developed for Ebola virus disease,^9^ can be modified and applied to the ED evaluation and management of patients presenting with symptoms suggestive of HAV infection (Figure). The first branch of the algorithm entails *identifying* suspected cases based on clinical signs/symptoms and exposure history. Of note, patients are contagious *prior* to symptom onset and some patients may never develop symptoms. In addition, a typical milder *relapsing hepatitis* may occur two weeks or more after initial symptom onset in approximately 2–20% of patients (10% in the 2017 San Diego outbreak) making it important to query patients about whether their symptoms are recurrent.

As transmission of HAV is mainly fecal-oral, patients require standard and enteric precautions, but airborne and respiratory droplet *isolation* precautions are not required. Blood samples should be obtained from patients with suspected HAV to confirm the diagnosis. Providers caring for patients with suspected HAV infection should observe strict standard precautions and hand hygiene with soap and warm water in all cases. Healthcare providers should ensure to wash hands for at least 10–20 seconds.^46^,^50^ Healthcare workers should additionally use contact precautions when caring for incontinent or diapered patients.

Patients should be counseled on the importance of hand hygiene. Affected patients should also be instructed to avoid food preparation for others and patients who work in food service, health service or in child care facilities should be advised to avoid work until two weeks after onset of initial symptoms or jaundice (whichever occurs later). Patients presenting within two weeks of exposure should be offered PEP (vaccination or IG as appropriate, based on age and comorbidities). A healthcare advisory released July 20, 2017, followed by a CDC publication on September 15, 2017, recommended an increase in the dosage for IG for pre- and post-exposure prophylaxis.^42^

Healthcare providers should promptly *inform* the local public health department of suspected and confirmed cases of HAV. Timely notification is particularly important when homeless and illicit drug users are affected as these patient populations can be difficult to trace once discharged from the ED. Providers should also notify hospital infection control personnel of suspected cases and abide by any additional legal requirements such as notification of exposed prehospital personnel.^43^,^51^

Because vaccination is a key component of outbreak control, some public health experts have recommended adding another “I” to the algorithm, specifically to represent “immunize.”^52^ Immunization of at-risk populations who present to the ED for unrelated reasons is an important public health intervention.^8^ To assist providers with remembering to vaccinate, the “Identify” branch of the 3I algorithm can be thought of as “Identify/Immunize” for management of both infected and vulnerable populations. Of note, it takes approximately two weeks for immunity to develop after vaccination administration.

## CONCLUSION

HAV is a highly contagious viral disease with the potential for severe morbidity and mortality. Although the overall incidence of the disease has been decreasing in developed countries since the development of the HAV vaccine, there have been a number of large outbreaks in several U.S. states and elsewhere. The Identify-Isolate-Inform tool will serve as a useful instrument for ED providers to apply in the evaluation and management of patients who present with possible HAV exposure or infection.

## Figures and Tables

**Figure:**
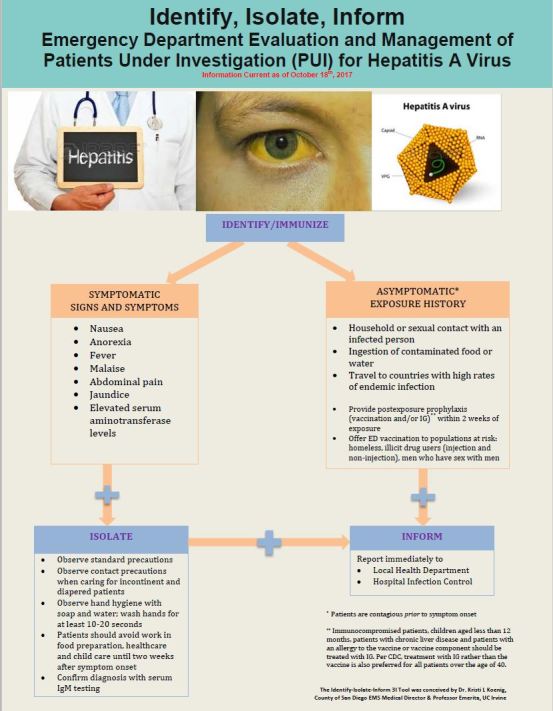
Identify-Isolate-Inform tool adapted for Hepatitis A virus.

## References

[1] Ly KN, Klevens RM (2015). Trends in disease and complications of Hepatitis A virus infection in the United States, 1999–2011: a new concern for adults. J Infect Dis.

[2] Wasley A, Samandari T, Bell BP (2005). Incidence of Hepatitis A in the United States in the era of vaccination. JAMA.

[3] Hepatitis A Southeast Michigan Outbreak.

[4] Hepatitis A (2017). San Diego Hepatitis A Outbreak.

[5] (2016). Hepatitis A, Outbreak.

[6] Outbreaks of Hepatitis A in Hawaii linked to raw scallops.

[7] 2016 - Multistate outbreak of hepatitis A linked to frozen strawberries.

[8] James TL, Aschkenasy M, Eliseo LJ (2009). Response to hepatitis A epidemic: emergency department collaboration with public health commission. J Emerg Med.

[9] Koenig KL (2015). Identify, Isolate, Inform: A 3-pronged Approach to Management of Public Health Emergencies. Disaster Med Public Health Prep.

[10] Koenig KL, Burns MJ, Alassaf W (2015). Identify-Isolate-Inform: a tool for initial detection and management of measles patients in the emergency department. West J Emerg Med.

[11] Koenig KL (2015). Identify-Isolate-Inform: A modified tool for initial detection and management of Middle East Respiratory Syndrome patients in the emergency department. West J Emerg Med.

[12] Koenig KL, Shastry S, Mzahid B (2016). Mumps virus: modification of the identify-isolate-inform tool for frontline healthcare providers. West J Emerg Med.

[13] Lednar WM, Lemon SM, Kirkpatrick JW (1985). Frequency of illness associated with epidemic HAV virus infections in adults. Am J Epidemiol.

[14] Cuthbert JA (2001). HAV: old and new. Clin Microbiol Rev.

[15] Tong MJ, el-Farra NS, Grew MI (1995). Clinical manifestations of HAV: recent experience in a community teaching hospital. J Infect Dis.

[16] Inman RD, Hodge M, Johnston ME (1986). Arthritis, vasculitis, and cryoglobulinemia associated with relapsing Hepatitis A virus infection. Ann Intern Med.

[17] Dan M, Yaniv R (1990). Cholestatic hepatitis, cutaneous vasculitis, and vascular deposits of immunoglobulin M and complement associated with HAV virus infection. Am J Med.

[18] Schiff ER (1992). Atypical clinical manifestations of Hepatitis A. Vaccine.

[19] Ilan Y, Hillman M, Oren R (1990). Vasculitis and cryoglobulinemia associated with persisting cholestatic Hepatitis A virus infection. Am J Gastroenterol.

[20] Lavine J, Bull F, Millward-Sadler G, Millard-Sadler G, Wright R, Arthur M (1992). Acute viral hepatitis. Wright’s Liver and Biliary Disease.

[21] Shenoy R, Nair S, Kamath N (2004). Thrombocytopenia in Hepatitis A--an atypical presentation. J Trop Pediatr.

[22] Update #3: Hepatitis A virus outbreak in San Diego County.

[23] Daniels D, Grytdal S, Wasley A, Centers for Disease Control and Prevention (CDC) (2009). Surveillance for acute viral hepatitis - United States, 2007. MMWR Surveill Summ.

[24] Klevens RM, Miller JT, Iqbal K (2010). The evolving epidemiology of Hepatitis A in the United States: incidence and molecular epidemiology from population-based surveillance, 2005–2007. Arch Intern Med.

[25] (2017). Epidemiological update: hepatitis A outbreak in the EU/EEA mostly affecting men who have sex with men.

[26] Gozlan Y, Bar-Or I, Rakovsky A (2017). Ongoing Hepatitis A among men who have sex with men (MSM) linked to outbreaks in Europe in Tel Aviv area, Israel, December 2016 – June 2017. Euro Surveill.

[27] Bohm SR, Berger KW, Hackert PB (2015). Hepatitis A outbreak among adults with developmental disabilities in group homes--Michigan, 2013. MMWR Morb Mortal Wkly Rep.

[28] Latash J, Dorsinville M, Del Rosso P (2017). Notes from the field: increase in reported hepatitis A infections among men who have sex with men – New York City, January–August 2017. MMWR Morb Mortal Wkly Rep.

[29] Nainan OV, Xia G, Vaughan G (2006). Diagnosis of Hepatitis A virus infection: a molecular approach. Clin Microbiol Rev.

[30] Ajmera V, Xia G, Vaughan G (2011). What factors determine the severity of Hepatitis A-related acute liver failure?. J Viral Hepat.

[31] Manka P, Verheyen J, Gerken G (2016). Liver failure due to acute viral hepatitis (A–E). Visc Med.

[32] Squires RH, Shneider BL, Bucuvalas J (2006). Acute liver failure in children: the first 348 patients in the pediatric acute liver failure study group. J Pediatr.

[33] Ciocca M, Moreira-Silva SF, Alegría S (2007). Hepatitis A as an etiologic agent of acute liver failure in Latin America. Pediatr Infect Dis J.

[34] Ciocca M, Ramonet M, Cuarterolo M (2008). Prognostic factors in paediatric acute liver failure. Arch Dis Child.

[35] Kemmer NM, Miskovsky EP (2000). Hepatitis A. Infect Dis Clin North Am.

[36] Vento S, Garofano T, Renzini C (1998). Fulminant hepatitis associated with hepatitis A virus superinfection in patients with chronic hepatitis C. N Engl J Med.

[37] Gordon SC, Reddy KR, Schiff L (1984). Prolonged intrahepatic cholestasis secondary to acute HAV. Ann Intern Med.

[38] Jung YM, Park SJ, Kim JS (2010). Atypical manifestations of Hepatitis A infection: a prospective, multicenter study in Korea. J Med Virol.

[39] Adhami T, Hannouneh I, Hepatitis A (2014). Cleveland Clinic Center for Continuing Education: Disease Management.

[40] Tabak F, Ozdemir F, Tabak O (2008). Autoimmune hepatitis induced by the prolonged Hepatitis A virus infection. Ann Hepatol.

[41] Hepatitis A and Food Service Workers (infectious hepatitis).

[42] CDC (2017). Updated Dosing Instructions for Immune Globulin (Human) GamaSTAN S/D for Hepatitis A Virus Prophylaxis Weekly / September 15, 2017. Morb Mortal Wkly Rep (MMWR).

[43] Hepatitis A Questions and Answers for Health Professionals.

[44] California Department of Public Health – July 2017: Hepatitis A Postexposure Prophylaxis Guidance.

[45] AFSCME Health & Safety Fact Sheet: Hepatitis A.

[46] Hepatitis A Infection Prevention and Control.

[47] Matheny SC, Kingery JE (2012). Hepatitis A. Am Fam Physician.

[48] Gilroy RK Hepatitis A. Medscape.

[49] Prevention of Hepatitis A Through Active or Passive Immunization Recommendations of the Advisory Committee on Immunization Practices (ACIP).

[50] Clean Hands Count for Healthcare Providers.

[51] Update #4: Hepatitis A Virus Outbreak in San Diego County.

[52] (2017). Personal communication with Eric C. McDonald, MD, MPH, FACEP, Medical Director, Epidemiology & Immunization Services Branch, County of San Diego, Health & Human Services Agency.

